# Polypharmacy in a hospitalized psychiatric population: risk estimation and damage quantification

**DOI:** 10.1186/s12888-019-2056-0

**Published:** 2019-02-21

**Authors:** J. Carmona-Huerta, S. Castiello-de Obeso, J. Ramírez-Palomino, R. Duran-Gutiérrez, D. Cardona-Muller, F. Grover-Paez, P. Fernández-Dorantes, R. Medina-Dávalos

**Affiliations:** 1 Instituto Jalisciense de Salud Mental, Avenida Zoquipan 1000-A, Zip code 45170 Zapopan, Jalisco México; 2 Centro Universitario de Ciencias de la Salud, Universidad de Guadalajara, Sierra mojada 950 Colonia independencia, Zip code 44340 Guadalajara, Jalisco México; 3 Departament of Experimental Psychology, Oxford University, Guadalajara, Mexico; 4 ITESO, Univesidad Jesuita de Guadalajara, Sierra mojada 950 Colonia independencia, Zip code 44340 Guadalajara, Jalisco México

**Keywords:** Polypharmacy, Antipsychotic, Secondary effect, Extrapyramidal symptoms, Hospitalized psychiatric population, Pharmacological interactions, Drug-drug interaction, Antipsychotic prescription duplicity

## Abstract

**Background:**

Polypharmacy increases the risk of pharmacological interactions, prevalence of secondary effects and with this the lack of adherence to treatment. It is estimated that between 10 and 40% of patients hospitalized in psychiatric institutions are prescribed more than one antipsychotic. The objective of the present study was to identify the prevalence of polypharmacy, evaluate adverse effects associated to the use of psych drugs and to estimate the risk in specific groups.

**Methods:**

We carried out a longitudinal, retrospective study that included the analysis of all discharged patients (*n* = 140) in the first trimester of the year in a psychiatric hospital in Mexico. The information was classified into 7 sections: sociodemographic, diagnosis, clinical follow-up information, prescribed drugs, adverse reactions, substance abuse, laboratory and complementary results.

Risk estimation was obtained with Odds Ratios, to correlate continuous variables Pearson’s correlation was used. Student’s T and Mann Whitney’s U were used to compare 2 independent samples; multiple and linear regressions were carried out.

**Results:**

The mean number of drugs used during hospitalization was 7.8 drugs per patient. The mean prescribed psych drugs was 4.07. The mean antipsychotic dose was the risperidone equivalent of 5.08 mg. 29.2% of patients had at least one secondary effect associated to the use of drugs, 17.8% presented extrapyramidal symptoms. 81.4% of patients were prescribed 6 or more drugs (polypharmacy) and were 5 times more likely to suffer a secondary effects (OR 6.24). 14.2% had polypharmacy while receiving antipsychotics and had more than twice the risk of presenting extrapyramidal symptoms (OR 3.05). For each added psych drug, hospital stay increased by 6.56 days.

**Conclusions:**

Despite international guideline recommendations where reasoned and conciliatory prescription of psych drugs is advised, there is still a high prevalence of polypharmacy in patients hospitalized in psychiatric institutions. In the present study 4 out of 5 patients received polypharmacy decreasing tolerability, treatment adherence and increasing the risk and costs secondary to an increased hospital stay.

## Background

In psychiatric clinical practice interactions increases with the number of drugs used. Despite current international guidelines recommendations, the administration of several psychodrugs; defined as drugs with effects in CNS administered with the purpose of treating mental illness; in patients admitted in psychiatric institutions is commonly encountered. In fact, there is a high prevalence of polypharmacy (the continued use of any 6 or more enteral or parenteral drugs during a period of at least 2 weeks). In these patients, the prevalence of adverse effects secondary to pharmacological interactions between psychodrugs and also between psychodrugs and other non-psych drugs is close to 90% [1]. The prescription of two or more antipsychotics and the combination of a SSRI and a TCAs, because of their pharmacokinetic and pharmacodynamics properties, have an especially high risk of presenting drug-drug interactions. This in turn poses a risk compromising safety and tolerability of the ongoing treatment. One of the most common presentations of these interactions are adverse events, generating repercussions for future pharmacologic adherence [2].

On the other hand, despite the lack of indications, it is common to see the use of two or more antipsychotic agents (duplicity) [3, 4]. In fact, it is estimated that between 10 and up to 40% of hospitalized patients receive two or more antipsychotics [5, 6].

For this reason, it is imperative to investigate the effects and repercussions of this off-label prescription habit in a mental health institution that represents Mexican psychiatric practice. The objective of the present study was to obtain a situational diagnosis regarding drug use, frequency of use, polypharmacy prevalence, risk estimation for specific groups and the damage quantification determined through the evaluation of adverse effect presentation (including extrapyramidal effects) associated to their use.

## Methods

### Study design

We reviewed all the 2017 first trimester patient discharge files in the prolonged stay department of the CAISAME hospital, the largest psychiatric hospital of Mexico’s western region, which has 252 available hospitalization beds. This is a mental illness referral centre that treats severe chronic psychiatric disorders, and offers its services to a referral population of roughly 10 million persons in an area equivalent to the country of Portugal, 3 times the size of Massachusetts [7].

In the period between January the first and March 31st 2017 there were a total of 140 patient discharge files. The review of these files included the creation of a special pharmacovigilance format Pharmacovigilance format of the CAISAME Prolonged Stay Hospital stay patients (FHCEP, from its Spanish acronym). This institution admits patients for periods of approximately 45 days. This format was specifically made for this investigation and used to gather information regarding: socio-demographic, clinical and pharmacologic information, in order to evaluate the quality of attention and to develop an investigation that favours the improvement of clinical practices.

The information included in the FHCEP was organized in 7 sections: socio-demographic, diagnostic, clinical follow-up information, prescribed drugs, adverse reactions, substance abuse, and laboratory results. From this format information was extracted and captured in a database for latter analysis. All the information obtained was processed in a way that guaranteed the patients privacy and anonymity.

This retrospective study followed the recommendations and guidelines established in the Helsinki declaration and its four major principles: beneficence, non-maleficence, justice and autonomy. It was approved by the ethics and investigation committee of the Instituto Jalisciense de Salud Mental (Jalisco’s Mental Health Institute). The patients signed an informed consent form allowing the inclusion of their data in the present study and its publication.

### Procedure

Two trained physicians were responsible of reviewing the discharge files and of the FHCEP format generation. They did not participate in database data capture or data analysis, this was done to guarantee patient anonymity and to avoid result bias.

FHCEP format completion was done in a systematic manner, in order to avoid missing data, the information was not saved unless all the required information was included. Socio-demographic information included; age, gender, marital status, number of school years, occupation, place of residence, in-hospital time (days) and number of previous hospitalizations. The diagnostic section included up to 4 psychiatric disorders and 15 comorbid medical conditions. Section 3, clinical follow-up information, included: reason for discharge, height, weight, BMI at the moment of hospitalization and discharge, tobacco use, falls during hospital stay, reported events (insomnia, agitation, auto/hetero-aggression, suicidal attempts, disorganized behaviour, escape attempts), physical restraint requirement, and time to first psychotic episode occurrence (if applicable). Section 4, prescribed drugs, was divided in two parts, psychiatric agents, that included 5 different antipsychotic agents, 4 mood stabilizing drugs, 2 benzodiazepines, 2 antidepressants, 2 anticholinergics (for side effect treatment) and others. All drugs information included minimum and maximum dose. The second part of this section included general drugs and included anti-diabetic, antihypertensive, lipid lowering, NSAIDs, steroids, antibiotics, prokinetics/antacids and “other” agents. Section 5, adverse reactions, included: extrapyramidalism (akathisia, acute dystonia, Parkinsonism, tardive dyskinesia) as well as other drug related side effects like, ataxia, excessive somnolence, seizures, sialorrhea, vertigo/dizziness, nausea/vomit, exanthematic reactions, photosensitivity, amenorrhea, galactorrhea, gynecomastia and “others”. Section 6, drug abuse, registered substance abuse 6 months prior to admission (Alcohol, cannabis, methamphetamines, cocaine, inhaled agents, hallucinogens, opioids, and un-prescribed benzodiazepines and anticholinergics); drug abuse was considered only when the agents were not indicated for medical purpose. Finally section 7 included laboratory and imaging results at admission and discharge; complete blood count, blood chemistry, electrolytes, hepatic function, cholesterol, triglycerides, prolactin, and, if required, lithium and valproate serum levels.

### Measurement and categorization

Polypharmacy was defined as the continued use of any 6 or more enteral or parenteral drugs during a period of at least 2 weeks during hospitalization. Prescription duplicity was defined as the continued use of 2 or more antipsychotics for at least 2 weeks (without considering the cases where the intention for the second antipsychotic was to substitute the initial one). Secondary effect was considered as the presence of an adverse reaction related to drug use, and was included for analysis if there was a registry of this event in the clinical file or quantified through the Secondary effects scale UKU (for its German acronym Udvalg Fur Kliniske Undersogelser) [8]. The presence of akathisia, acute or recent onset dystonia, parkinsonism, late tardive dyskinesia was considered extrapyramidalism and was included for analysis if there was a registry of this event in the clinical file and quantified through the Simpson-Angus for parkinsonism Scale [9], BARNES for akathisia [10] and AIMS for dystonia or late tardive dyskinesia [11]. Substance abuse was considered for analysis if it was documented in the clinical file and if it happened within 6 months of admission. It was also considered in the case of a positive response of question 2 in the ASSIST scale [12].

Use of a “Psych Drug” was considered as the use of any of the following: antipsychotics, antidepressants, anxiolytics, mood stabilizers and antiparkinsonism agents.

To obtain the equivalent dose of Chlorpromazine we used Andreasen and Cols.’s linear regression formula. We calculated a dose equivalent to 100 mg of Chlorpromazine of the diverse first and second-generation antipsychotic agents. Al relationships were linear and had R^2^ > 0.9 [13].

For the description of the main psychiatric diagnoses we used the 10th version of the international disease classification (CIE 10) [14]. This same classification was also used for non-psychiatric diseases [15].

Described sociodemographic variables were: gender, marital status, occupation, place of residence, age, schooling years, number of previous hospitalizations, and days of hospital stay.

### Statistical analysis

Sociodemographic, ordinal, and nominal (diagnosis, comorbidities, substance abuse and used pharmacologic groups) variables are presented in frequencies and percentages; continuous variables (age, education years, number of hospitalizations, hospital stay (days), number of drugs used, and dose are shown in means and standard deviations. Risk was estimated with Odds Ratios (OR) with a 95% confidence interval. For difference determinations statistical significance was accepted if *p* <  0.05.

The correlation of continuous variables was performed with Pearson’s R. Multivariate analysis was done with one-way ANOVA if the sample had a normal distribution and equal variances and homoscedasticity, both tests with α = 0.05. To evaluate normality Shapiro Wilk’s test was used and for homoscedasticity Levene’s test. If any of these tests didn’t meet assumptions, we used Kruskal Wallis. To compare means we used Student’s T and if the sample didn’t meet normality or homoscedasticity criteria, Mann Whitney’s U was used. For all hypothesis contrasts and correlations α = 0.05 was used.

Multiple regression was used to predict hospital stay (days) as the dependent variable which was denoted as *y.* For this we introduced four independent variables. Statistical significance was assumed with a confidence interval (CI) of 95% and α = 0.05. Multiple regression was calculated with the following equation:

1 $$ y={\beta}_0+{\beta}_1{x}_1+{\beta}_2{x}_2+{\beta}_3{x}_3+{\beta}_4{x}_4 $$

Where x_1_ = equivalence to risperidone, x_2_ = number of psych drugs, x_3_ = number of consumed illegal substances y x_4_ = quantity of diagnoses. Interaction between independent variables was not explored. To assess multicollinearity, we obtain the Variance Inflation Factors (VIF). If the VIF is > 10, it suggests a high degree of multicollinearity, but > 2 has been suggested and used as a cutoff [16].

The analysis was carried out with the programs Statistical Package for the social sciences SPSS and “RStudio” which uses R language developed and updated by “The R Project for Statistical Computing” [17].

## Results

### Sociodemographic

The study included 140 subjects, 65% male, the average age was 34 years and 87% of subjects were unemployed. Regarding marital status, 74% of the patients were single, with schooling years mean of 7.5 years. The previous hospitalization mean was 2.2 and the mean hospital stay was 31.7 days for each hospitalization period. The rest of the variables are shown in Table 1.

**Table 1:** Descriptive sociodemographic variables. Main diagnosis of discharge, and comorbidities of discharge patients in the first trimester of the year (*n* = 140)

Variables	Categories	Frequency (%)
Gender	Female	49 (35)
	Male	91 (65)
Civil status	Single	104 (74)
	Married	17 (12)
	Separate	10 (7)
	Widower	9 (6)
Occupation	Employee	18 (13)
	Unemployed	122 (87)
Residency	Metropolitan zone	76 (54)
	Country	46 (33)
	Other states	18 (13)
Diagnosis	Schizophrenia	65 (46.4)
	Substance-induced psychotic disorder	26 (18.6)
	Type 1 bipolar	18 (12.9)
	Intellectual disability	7 (5)
	Psychotic disorder due to medical illness	5 (3.6)
	Mayor depression disorder	4 (2.9)
	Personality disorder	2 (1.4)
	Schizoafective disorder	2 (1.4)
	Other	11 (7.9)
Comorbidities	Consumption of substances (except tobacco)	89 (63.6)
	Overweight or obesity	67 (47.9)
	Smoking	53 (37.9)
	Dyslipidemias	43 (30.7)
	Arterial hypertension	13 (9.3)
	Mellitus diabetes	9 (6.4)
	Categories	Mean (SD)
	Age (in years)	34 (11.2)
	Scholarship (in years)	7.5 (3.3)
	Number of hospitalizations	2.2 (1.9)
	Days of hospital stay	31.7 (24)

Note: *SD* Standard Deviation

The most common discharge diagnoses were schizophrenia (46.4%), substance induced psychotic disorder (18.6%), and bipolar disorder (12.9%). Together these diseases represented 78% of all psychiatric disorders included; the rest of diagnoses are shown in Table 1. Only one psychiatric diagnosis was present in 77.85% of discharges (*n* = 109), and the rest of the patients had 2 or more diagnoses at the time of discharge.

### Substance abuse and comorbidities

The most common comorbidities were: substance abuse (including alcohol, illegal drugs and non-prescribed psychotropic medication, tobacco abuse was analysed separately) with a 63.6% prevalence within 3 months of admission, 47.9% suffered from overweight or obesity, tobacco use was seen in 37.9%, and lipid disorders in 30.7% of the sample. The 3 most commonly consumed substances within 3 months of admission were: ethanol (43.6% of cases), cannabis (41.4%) and methamphetamines (34.3%). The mean number of substances consumed was 2.48 ± 1.2 (*n* = 89). It is worth mentioning that in subjects with substance abuse, the main diagnosis was secondary to this abuse in 18.6%, (*n* = 26; drug induced psychotic disorder) while the other 63 cases had some other main diagnoses and substance abuse was an added psychiatric diagnosis (Table 2).

**Table 2:** Substances consumed in the last 3 months and number of substances consumed per patient (*n* = 140)

Substance	Frequency (%)
Alcohol	61 (43.6)
Cannabis	58 (41.4)
Methamphetamines	48 (34.3)
Cocaine	25 (17.9)
Inhalants	23 (16.5)
Benzodiazepine	5 (3.6)
Opioids	1 (0.7)
Number of substances consumed	
Without consumption	51 (36.4)
One	27 (19.3)
Two	20 (14.3)
Three	19 (13.6)
Four	19 (13.6)
Five	3 (2.1)
Six	1 (0.7)
	Mean (SD)
Mean of substances consumed	2.48 (1.2)

### Drug use and polypharmacy

The mean of the total number of drugs used per patient during hospitalization was 8.64 ± 4.13, and without taking into account the drugs used for incidences the mean was 7.88 ± 3.4. With respect to psych drugs, the mean number of medications during hospitalization was 4.07 ± 1.88. The frequency of prescription was the following: at least one antipsychotic (98.5%), benzodiazepines (90%), mood stabilizing agents (41.4%), antidepressants (27.8%) and antiparkinsonism (13.5%).

Although at the moment of discharge 138 out of 140 patients had a prescribed antipsychotic, without taking into account incidents and isolated dosing, the total amount of prescribed antipsychotics was 288, this was due to changes in prescribed dopaminergic blocking antipsychotics (switching). These changes in prescription were done as follows; 91 had a once only change, 41 had a change in the prescribed antipsychotic twice, 11 on 3 occasions and 7 on 4, no patient had his antipsychotic treatment changed for a 5th time.

The most commonly prescribed antipsychotic agents at discharge were: Risperidone 33.6%, Haloperidol 30% (haloperidol was prescribed both in its oral presentation,12.9% and as a parenteral depot antipsychotic, 17.1%) and Olanzapine (22.9%). These 3 drugs represent 86.5% of the total prescription of dopaminergic blockers. 20.6% of the subjects were discharged with a deposit antipsychotic agent and the mean dose of antipsychotics used during hospitalization was 5.08 mg represented according to their risperidone equivalence.

The presence of secondary effects associated to the use of drugs was reported as follows: 29.2% (*n* = 41) of patients had at least one secondary effect, 17.8% (*n* = 25) were extrapyramidal effects (EPE), 15% (*n* = 21) had non EPE secondary effects and 5 subjects had both EPE and non EPE secondary effects (Table 3).

**Table 3:** Characteristics of the prescribed drugs

Drugs	Mean (SD)
General drugs	3.87 (2.3)
Psychotropic drugs	4.07 (1.9)
Total drugs (no incidences)	7.88 (3.4)
Total drugs (incidents included)	8.64 (4.1)
Dosage of equivalent antipsychotic in milligrams of risperidone	5.08 (2.97)
Amount of prescribed drugs	Frequency (%)
Less than 6 drugs	26 (18.6)
6 or more drugs	114 (81.4)
Psychotropic drugs	Frequency (%)
Antipsychotic (AP)	138 (98.5)
Benzodiazepines	126 (90)
Mood stabilizer	58 (41.4)
Antidepressant	39 (27.8)
Antiparkinsonian	19 (13.5)
Antipsychotic type	Frequency (%)
Risperidone	47 (33.6)
Olanzapine	32 (22.9)
Haloperidol decanoate	24 (17.1)
Haloperidol	18 (12.9)
Aripiprazol	5 (3.6)
Clozapine	3 (2.1)
Deposit Zuclopenthixol	3 (2.1)
Deposit Risperidone	2 (1.4)
Quetiapine	2 (1.4)
Zuclopentixol	1 (0.7)
Trifluoperazine	1 (0.7)
Antipsychotic combination and side effects	Frequency (%)
Polypharmacy with antipsychotics	20 (14.2)
Side effects due to the use of drugs	41 (29.2)
Presence of extrapyramidal effects	25 (17.8)

### Predictive analysis

Due to the importance of the outcome variable “days of hospital stay” has, we undertook a series of analysis between this and the other independent and intervening variables.

**Fig. 1:**
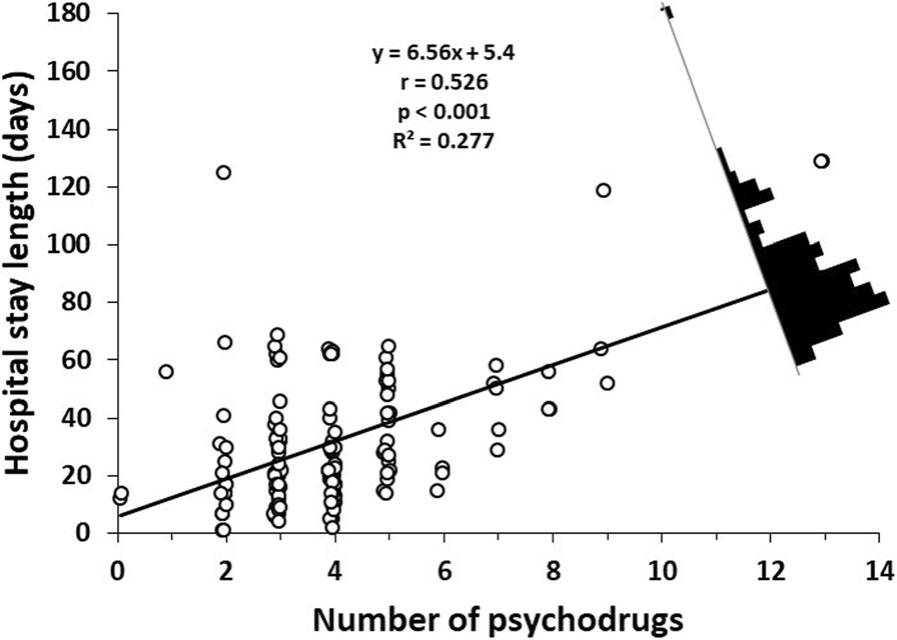
Linear regression of “Hospital stay length (days)” and “Number of psychodrugs”. The residual distribution is represented by the black histogram. In the superior central white area it is presented the linear regression function, the correlation (*r*), *p* value for the significant test of the correlation and the determination coefficient (*R*^*2*^)

There was a positive relation between the number of prescribed drugs and “days of hospital stay” with the correlation coefficient [*r*(138) = 0.5362, *t* = 7.27, *p* <  0.001] for both variables, we then performed a simple linear regression (Fig. 1) where we observed that for each prescribed psych drug, hospital stay increased 6.56 days (slope of the linear equation). This was consistent with the coefficients shown in the multiple regression (Table 4). Statistical significance was obtained for the variable “Number of psych drugs (β_2_)” with a coefficient of 6.67, which, assuming the other variables are constant, would translate as a 6.67 day increase in hospital stay for each prescribed psych drugs. This multiple linear regression (Table 4), and the fit from the 11 models presented in the Table 5 allowed us to discard the influence (β_1_, β_3_ and β_4_) of the rest of the independent variables to predict “Hospital stay length (days)”.

**Table 4:** Al combination of multiple regression models with 2, 3 or 4 independent variables and their R^2^ with and without interaction included

	Independent variables				R^2^	
Models	β_1_	β_2_	β_3_	β_4_	No interaction	Interaction
1	Equivalence to Risperidone	Number of psychodrugs	NA	NA	0.277	0.283
2	Equivalence to Risperidone	NA	Illicit drugs	NA	0.003	0.004
3	Equivalence to Risperidone	NA	NA	Amount of diagnostics	0.001	0.009
4	NA	Number of psychodrugs	Illicit drugs	NA	0.281	0.294
5	NA	Number of psychodrugs	NA	Amount of diagnostics	0.279	0.297
6	NA	NA	Illicit drugs	Amount of diagnostics	0.003	0.073
7	Equivalence to Risperidone	Number of psychodrugs	Illicit drugs	NA	0.282	0.370
8	Equivalence to Risperidone	Number of psychodrugs	NA	Amount of diagnostics	0.280	0.317
9	Equivalence to Risperidone	NA	Illicit drugs	Amount of diagnostics	0.003	0.081
10	NA	Number of psychodrugs	Illicit drugs	Amount of diagnostics	0.284	0.333
11	Equivalence to Risperidone	Number of psychodrugs	Illicit drugs	Amount of diagnostics	0.285	0.424

Note: each model [1 to 11] was run 2 times, one with interaction and the other without interaction

**Table 5:** Multiple linear regression results (Model 11, without interaction)

	Coefficients (β)	S.E.	T statistic	P value	95% CI	VIF
Intercept (β_0_)	7.628	6.812	1.12	0.265	−5.844 – 21.099	NA
Equivalence to Risperidone (β_1_)	−0.189	0.594	−0.317	0.751	−1.364 – 0.987	1.0009
Number of psychodrugs (β_2_)	6.678	0.917	7.286	< 0.001	4.865–8.491	1.0202
Illicit drugs (β_3_)	1.061	1.116	0.951	0.343	−1.146 – 3.267	1.0022
Amount of diagnostics (β_4_)	−2.76	3.861	−0.715	0.476	−10.396 – 4.877	1.0194

Note: multiple regression it was not calculated with interaction between independent variables. Determination coefficient (R^2^) = 0.285. *S.E.* Standard Error, *CI* Confidence Interval, *VIF* Variance Inflation Factor

We have run 11 multiple regression models (Table 4, Models 1 to 11), the models were the combinations of 4 independent variables in 2, 3 and 4 coefficients (β). As it can be seen the highest R^2^ is obtained when the independent variable “Number of psych drugs” is in the models. In the Table 5 we show the coefficients for the model 11 without interaction. The only significant coefficients is the “Amount of psychotropic drugs”. This supports that the only potential relationship between variables is the one between Number of psychodrugs and Hospital stay length (days).

**Fig. 2:**
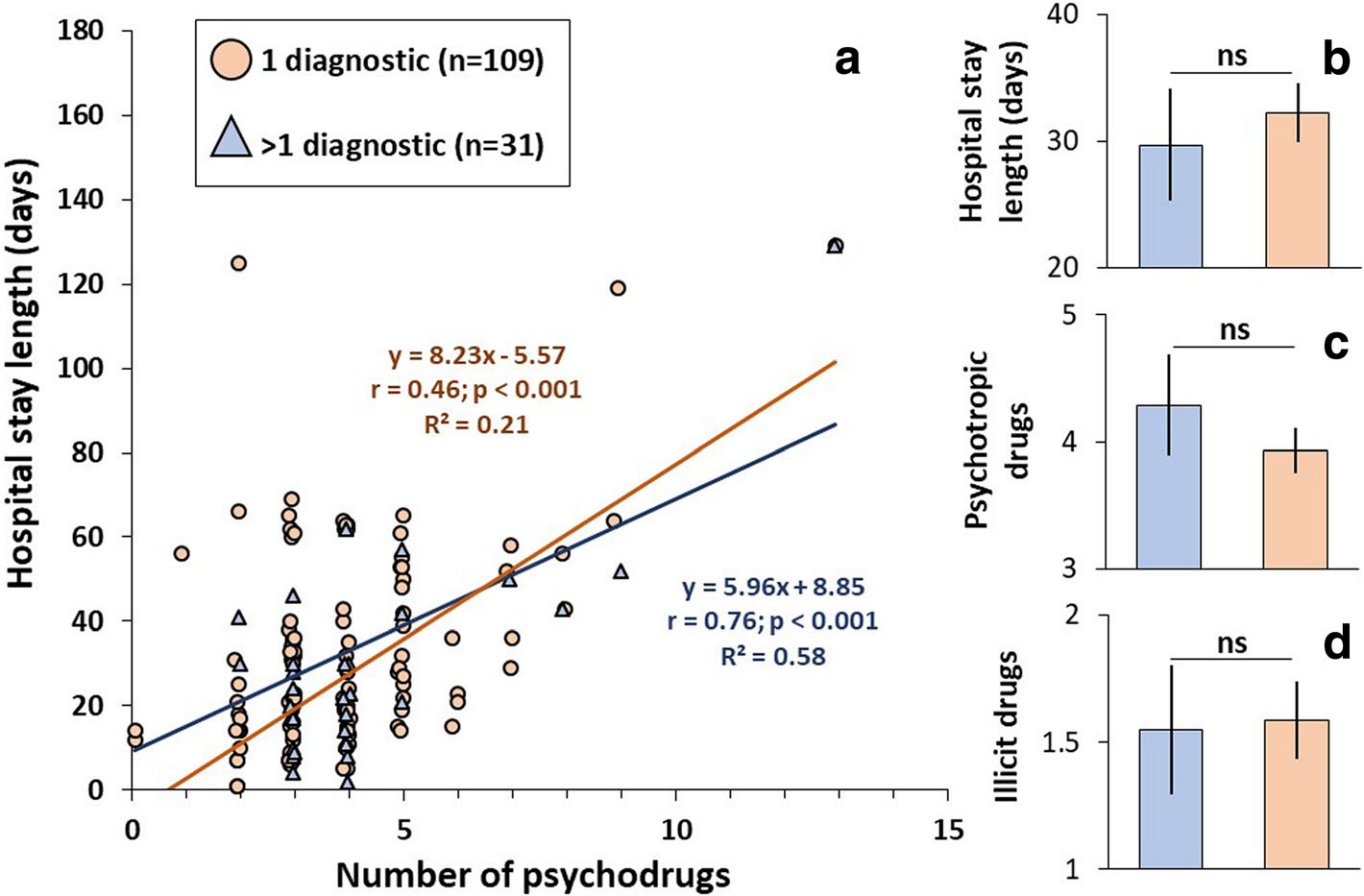
**a**. “Hospital stay length (days)” versus “Number of psychodrugs” for subgroups patients: “1 diagnostic” and “> 1 diagnostic”. The regression equations, correlations, *p* values and determination coefficient are presented in the correspondent colour for each subgroup. **b**, **c**, and **d**. Means and standard errors of hospital stay length, psychotropic drugs, and illicit drugs by subgroups. **ns** means no statistical significance by test Mann Whitney U

In this same line, and with the intention of the further understanding of the observed correlation, we divided the sample into 2 groups according to the presence of just 1 psychiatric diagnosis (*n* = 109) versus 2 or more diagnoses (*n* = 31). A statistically significant difference between correlations was found (see Fig. 2a): for “1 diagnosis”: *r*(92) = 0.4221, *t* = 4.466, *p* < 0.001; and for “> 1 diagnosis”: *r*(44) = 0.7453, *t* = 7.415, p < 0.001). The correlation in the group with > 1 psychiatric diagnosis was significantly greater (Fig. 2) [*r*_1 diagnosis_- *r*_> 1 diagnosis_ = − 0.422 - 0.745 = − 0.323; z = − 2.76; *p* = 0.0058]. After obtaining this result, we compared the variables “mean hospital stay”, “quantity of prescribed psych drugs” and “number of consumed illegal drugs” in the 3 months prior to hospitalization, between the subgroups “1 diagnosis” and “> 1 diagnosis”, (Fig. 2b, c and d). There was no statistically significant difference between these 3 variables (“days of hospital stay” U = 1811, *p* = 0.5435. “Number of psychodrugs” U = 1596.5, *p* = 0.6328. “consumed illegal drugs” U = 1650.5, *p* = 0.8418).

Concerning polypharmacy, 81.4% of the patients received 6 or more prescribed drugs, it was more than 6 times more likely to present a secondary effect if receiving 6 or more drugs vs 5 or less (OR 6.24, 95% IC 1.4 to 27.7, *p* < 0.005).

Finally, in the comparison between psych drug monotherapy for psychotic symptoms vs the use of 2 antipsychotics for this same purpose, 14.2% (*n* = 20) had prescription duplicity during hospitalization. This particular group had an increased risk of EPE compared to the group that received only 1 antipsychotic agent (OR 3.05, 95% IC 1.1 to 8.6, *p* < 0.05).

**Fig. 3:**
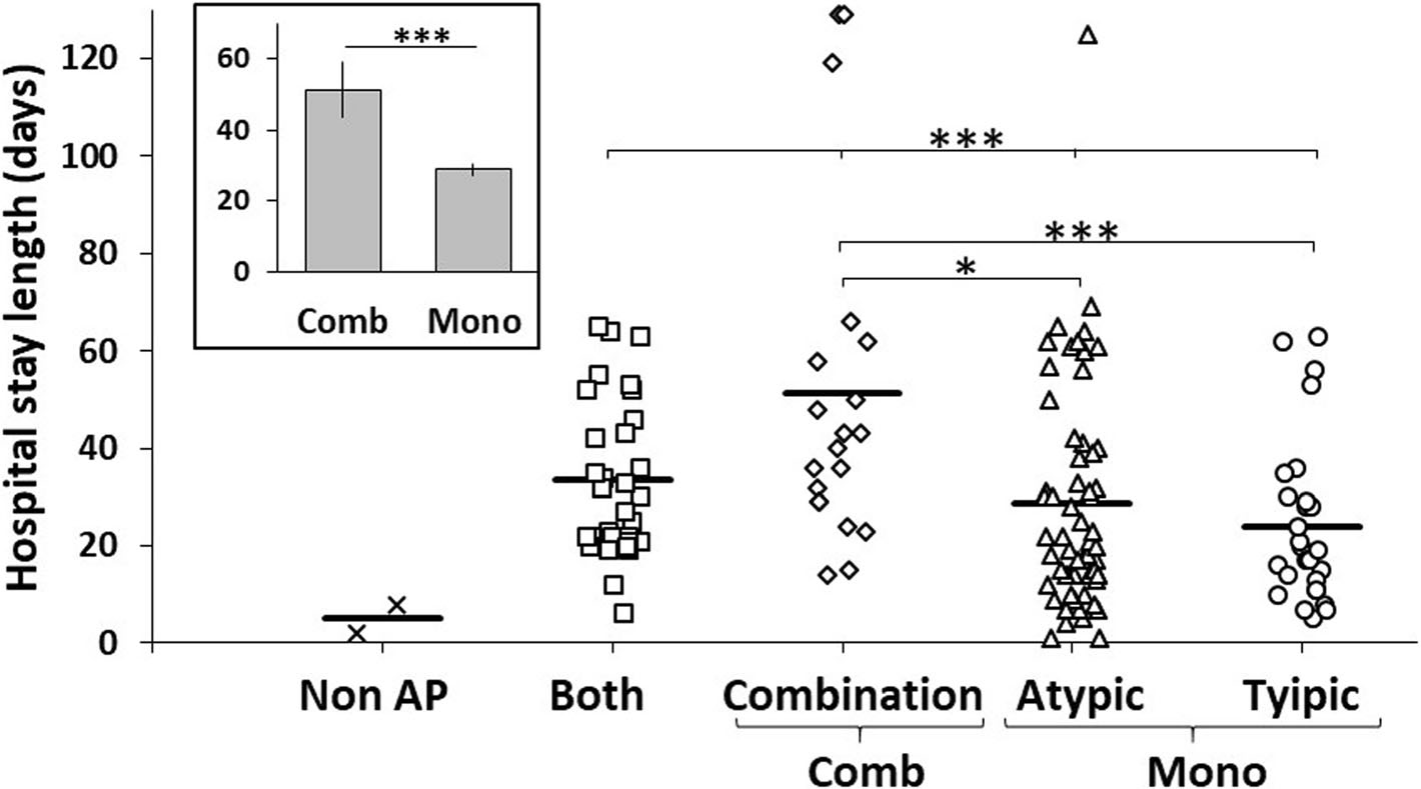
Five subgroups of patients with different treatments. Comparison for the 4 groups with Kruskal Wallis test. Post Hoc test by Nemenyi. Each symbol (cross, square, diamond, triangle or circle) represent one patient. Horizontal black tick lines show the mean group. Superior left bar plot shows mean and standard error for hospital stay length (days) by subgroups: Comb versus Mono (total patients of Atypical + Typical); Null hypothesis significant test was Mann Whitney U. *** represents *p* value < 0.001, ** *p* < 0.01 y * *p* < 0.05

An analysis was carried to compare the relationship between the different prescribed antipsychotics and “days of hospital stay” (Fig. 3). It was apparent that those with antipsychotic duplicity (under “combination” tag in the graph) tend to be the patients with longer hospital stays when compared with one those receiving one antipsychotic be it typical or atypical. There was no difference between those with prescription duplicity vs patients that received both typical and atypical antipsychotics without duplicity (those switching therapy after a failed therapeutic attempt).

To confirm the relationship between prescription duplicity and days of hospital stay we made a group that received just one type of antipsychotic (typical or atypical) and compared it with those with prescription duplicity. Once again, in a consistent manner, independent to the type of prescribed antipsychotic, patients treated with antipsychotic duplicity tend to have longer hospitalization periods (Annexed graph in Fig. 3).

## Discussion

Part of the richness of this study resides in the fact that the population represented is one that is usually found in countries with emerging economies (the presented results come from the second largest psychiatric hospital in the Mexico taking into account number of beds and the population assigned to it).

It is of note that the majority of admissions corresponds to young adults (mean age of 34 years) with severe and persistent mental disorders (schizophrenia, substance induced psychotic disorder and bipolar disorder), with an educational level that is barely above a basic one and in whom apparently, the functional status is already affected, this is inferred through the high unemployed proportion (74%).

The high prevalence of substance abuse is also a worrying fact, 63% of the sample reported the abuse of at least 1 substance (that wasn’t tobacco) 3 months prior to admission, of these sample, and more than 60% had consumed 2 or more substances. Substance abuse was the main admission diagnosis (substance induced psychotic disorder) in 1 out of 5 admissions. This fact shows a different trend regarding illegal substance consumption than what is currently described in Mexico’s 2011 [18] national addiction survey and even in the Alcohol, Tobacco and Drug abuse 2016 [19] survey, particularly with respect to methamphetamine consumption in this region. This is particularly alarming due to the fact that previously, in this particular region, methamphetamine consumption incidence was as low as 1.4% and the incidence reported for Mexico as a country is even lower (0.2%) [19]. Future studies that provide more evidence related to this phenomenon are needed to fully describe this potential shift in drug abuse pattern.

Regarding drug prescription, there was close an 80% polypharmacy incidence in this population. This is similar to what is reported [6] in other medical facilities similar to the one this study was conducted in. Nevertheless, this does not justify this practice. Most clinical prescription guidelines advice against the use of large numbers of pharmacologic agents and suggest a reasoned and conciliatory prescription. Polypharmacy is a disturbing phenomenon that led to an increased probability of presenting a secondary effect which was 6 times greater than in patients that received less than 5 drugs. There was an average 4 psych drugs indicated per hospitalized patient, increasing 6.56 hospitalization days per indicated drug, this relationship remained after a multiple regression was done in order to determine the possible influence of other intervening variables like different antipsychotic dose, substance abuse and number of diagnoses. This worrying finding demands the establishment of treatment algorithms and protocols in order to promote the proper diagnosis and treatment of this diseases with the hope of reducing the number of prescribed psych drugs and with this, possibly, the length of hospitalization and number of readmissions. This is supported by the study carried out by Baker and Cols. that reported an increased readmission rate in patients receiving polypharmacy when compared to those with monotherapy. One of the possible explanations for this phenomena is that in patients with polypharmacy adherence is much lower, in part secondary to the number of drugs taken daily and also to an increase in secondary effect incidence [20].

On the other hand, the high prevalence of antipsychotic use (98.5% had at least 1 prescribed antipsychotic) is secondary to the fact that the vast majority of the population admitted to the hospital has psychosis, and it is of note that one of the 2 most commonly prescribed antipsychotics given to control the symptoms associated with psychotic syndrome is a first generation drug. Most of the guidelines no longer include typical antipsychotic as first line agents [4]; one possible explanation for this prescription behaviour could reside in the acute and intense presentation of positive psychotic symptoms that required potent dopaminergic blockade, even taking into consideration the EPE risk increase. Adding to this, a significant number of these patients received antipsychotic duplicity (15% of the population) which had an even higher risk of presenting EPE and of having an increased hospital stay compared to those without duplicity (with either typical or atypical agents). Antipsychotic duplicity was associated with more than double the risk of presenting EPE and an increased hospital stay of an extra 20 days than those without duplicity [21, 22].

The duplicity prevalence seen in this study was similar to that previously reported [5, 6, 20–22] and gives account to off label practices carried out in multiple psychiatric hospitals from across de globe in the face of hospitalized acutely decompensated patients. Nevertheless, this behaviour remains controversial, most guidelines recommend the use of only 1 antipsychotic agent [3], but in light of this type of prescription behaviours, in a recently published Cochrane meta-analysis regarding antipsychotic use, there appears a possible new paradigm regarding the treatment of a specific population with psychosis. Of the analysed sub-groups, two had a better response to treatment with 2 antipsychotic agents [23]; one was a 17 study group that received clozapine plus another antipsychotic agent compared to the use of clozapine alone; the other was a 5 study subgroup that included typical dopaminergic blockers in both groups and reported a better response in the groups receiving two antipsychotics. The question remains taking into consideration our results and other evidence that is consistently replicated with respect to the negative effects of prescription duplicity with antipsychotics that is discordant to what was recently published in the previously mentioned meta-analysis regarding those two subgroups.

Some of the limitations in the present study were the inability to determine the presence of a pharmacological response in treated subjects or to estimate the clinical severity between the different subgroups (polypharmacy vs no polypharmacy, or antipsychotic duplicity vs monotherapy) due to the lack of indicators in all of the analysed files. Another limitation to consider in the present study was that the severity of symptoms was no evaluated and this could act as a confounding factor regarding the length of hospitalization. There is a need for prospective studies that analyse the effect of this factor on the evaluated patients.

## Conclusions

Although international guidelines have promoted for years a reasoned and conciliatory psych drug prescription, there is still a high prevalence of polypharmacy in patients hospitalized in psychiatric institutions with close to an 80% prevalence found in the present study. This in turn has severe implications regarding tolerability and the safety due to an increase in adverse events in these analysed patients suffering from mental disorders. On the other hand this same practice has implications regarding hospitalization costs with a 6 day increase per prescribed psych drug.

Antipsychotic duplicity is a poorly evidence supported practice, nevertheless, it is not uncommon to encounter patients receiving 2 antipsychotic agents, in our present study the prevalence for this practice was of 14.2%. This in turn was associated to a significantly increased risk of presenting EPE, drug-drug interaction and of having an increased hospital stay when compared to those receiving a single antipsychotic agent.

## Abbreviations

AIMS: Abnormal Involuntary Movement Scale
ANOVA: Analysis of Variance
AP: Antipsychotics
ASSIST: Alcohol, Smoking and Substance Involvement Screening Test
BMI: Body Mass Index
CAISAME: Centre of Integral Care in Mental Health
CI: Confidence Interval
CIE: International Disease Classification
CNS: Central Nervous System
EPE: Extrapyramidal Effects
FHCEP: Pharmacovigilance format of the CAISAME Prolonged Stay Hospital
ITESO: Institute of Technology and Higher Studies of the West
NSAIDs: Nonsteroidal anti-inflammatory drugs
OR: Odds Ratio
SD: Standard Deviation
SPSS: Statistical Package for the Social Sciences
SSRI: Selective Serotonin Re-uptake Inhibitor
TCA: Tricyclic Antidepressants
UKU: Udvalg Fur Kliniske Undersogelser
VIF: Variance Inflation Factor
